# A rare case of familial chylomicronemia in infancy due to homozygous *LMF1* variant

**DOI:** 10.1210/jcemcr/luag164

**Published:** 2026-10-05

**Authors:** Padala Ravi Kumar, Bandana Dash, Deepak Kumar Dash, Debasish Patro, Pradosh Kumar Sahu, Ankeet Biswas

**Affiliations:** Department of Endocrinology, MKCG Medical College, Berhampur, Odisha 760004, India; Department of Endocrinology, MKCG Medical College, Berhampur, Odisha 760004, India; Department of Endocrinology, MKCG Medical College, Berhampur, Odisha 760004, India; Department of Endocrinology, MKCG Medical College, Berhampur, Odisha 760004, India; Department of Endocrinology, MKCG Medical College, Berhampur, Odisha 760004, India; Department of Endocrinology, MKCG Medical College, Berhampur, Odisha 760004, India

**Keywords:** familial chylomicronemia syndrome, *LMF1* variants, lipemia retinalis

## Abstract

Familial chylomicronemia syndrome (FCS) is a rare autosomal recessive disorder of triglyceride (TG) metabolism, with an estimated prevalence of 1 in 100 000 to 1 in 1 000 000. It typically presents in childhood or adolescence, while presentation in infancy is exceedingly rare. Loss-of-function variants in lipoprotein lipase gene account for 60-80% of FCS cases. Other causes of FCS include loss-of-function variants in glycosylphosphatidylinositol-anchored high-density lipoprotein–binding protein 1, apolipoprotein A-V, apolipoprotein C-II, and lipase maturation factor 1 (*LMF1*) genes. *LMF1* variants are an exceedingly rare cause of FCS. We report a rare case of FCS presenting in a 5-month-old infant due to a homozygous variant in the *LMF1* gene, detected incidentally. Early diagnosis and timely dietary intervention resulted in significant improvement in serum TG levels and prevented complications like pancreatitis. This case highlights a rare presentation of FCS due to a homozygous *LMF1* variant presenting in infancy.

## Introduction

Hypertriglyceridemia is defined as serum triglyceride (TG) levels >100 mg/dL (>1.13 mmol/L) in children aged 0-9 years and >130 mg/dL (>1.47 mmol/L) in those aged 10-19 years. Severe hypertriglyceridemia is defined as TG levels >1000 mg/dL (>11.3 mmol/L) [1]. Most cases of hypertriglyceridemia in children and adolescents are secondary to obesity, insulin resistance, metabolic syndrome, or hypothyroidism [1]. Genetic causes of pediatric hypertriglyceridemia include familial chylomicronemia syndrome (FCS), transient infantile hypertriglyceridemia, multifactorial (polygenic) hypertriglyceridemia and lipodystrophy syndromes such as congenital generalized lipodystrophy, acquired generalized lipodystrophy, and familial partial lipodystrophy syndrome [2, 3].

FCS is a rare autosomal recessive disorder with an estimated prevalence of 1 in 100 000 to 1 in 1 000 000 [4]. FCS accounts for ∼1% of cases of severe hypertriglyceridemia [5]. FCS is a rare autosomal recessive monogenic disorder with high penetrance, caused by a biallelic loss-of-function variant in genes involved in the catabolism of circulating TG. Lipoprotein lipase (LPL) and its associated cofactors like apolipoprotein A-V (APOA5), apolipoprotein C-II (APOC2), glycosylphosphatidylinositol-anchored high-density lipoprotein–binding protein 1 (GPIHBP1) and lipase maturation factor 1 (LMF1) are involved in the catabolism of TG [5, 6]. The majority of cases of FCS (60-80%) are caused by biallelic *LPL* variants. More than 300 pathogenic or likely pathogenic *LPL* variants have been reported [6]. Other rare variants presenting as FCS include *GPIHBP1* (10-15%), *APOA5* (5-10%), *APOC2* (1-5%), and *LMF1* (1-2%) [6]. *LMF1* variants causing FCS are exceedingly rare.

*LMF1* encodes an endoplasmic reticulum protein essential for the proper folding, dimerization, and secretion of catalytically active lipases, like LPL, hepatic lipase, and endothelial lipase [5, 6]. Homozygous loss-of-function variants in *LMF1* result in combined lipase deficiency along with severe hypertriglyceridemia [5, 6]. Biallelic *LMF1* variants are a rare cause of FCS, with ages at presentation ranging from 23 to 65 years [5, 7-15]. We report a rare case of FCS due to a homozygous *LMF1* variant presenting in infancy, successfully managed with dietary modification. This case highlights a rare presentation of FCS due to a homozygous *LMF1* variant presenting in infancy. Infantile presentation of FCS is rare but clinically important because of the high risk of pancreatitis and long-term morbidity.

## Case presentation

A 5-month-old male infant presented to the pediatric emergency department with a history of a suspected snake bite on his back. There were no local or systemic signs of envenomation, and the clot retraction test was normal. However, blood samples drawn for evaluation showed a striking milky white appearance with a visible creamy supernatant within minutes of standing (Fig. 1A). He was managed conservatively and had no complications. Repeat blood samples drawn 2 weeks after discharge from the hospital showed persistence of the milky white appearance of serum (Fig. 1B).

**Figure 1:**
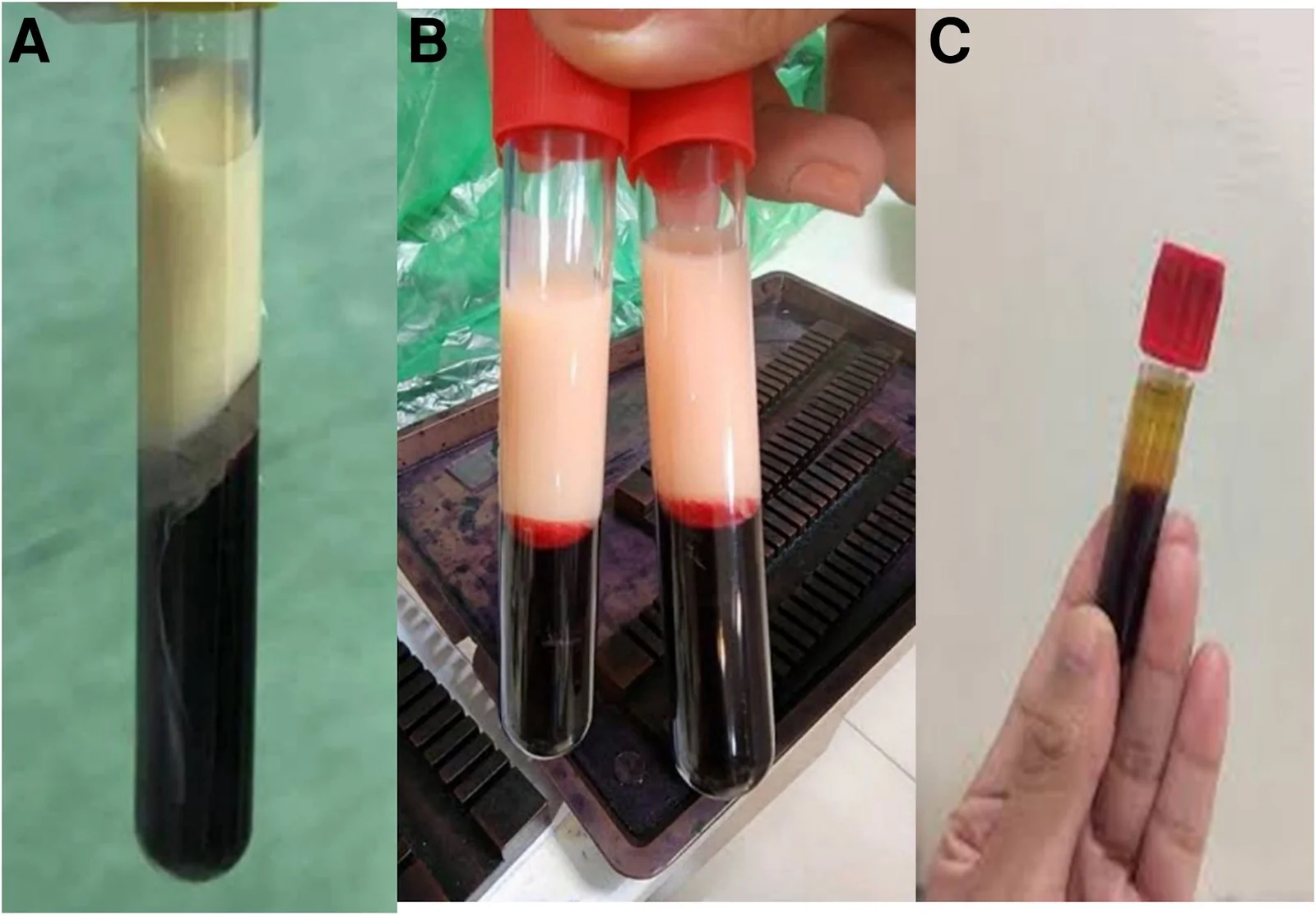
Sequential changes in serum appearance with dietary intervention. (A) Markedly lipemic serum at 5 months of age, corresponding to a serum triglyceride level of 8014 mg/dL (207.2 mmol/L). (B) Persistent lipemic serum at 6 months of age after 1 month of dietary intervention, with serum triglycerides reduced to 5040 mg/dL (56.9 mmol/L). (C) Nonlipemic serum after 5 months of dietary intervention, corresponding to a serum triglyceride level of 307 mg/dL (3.5 mmol/L).

The child was the first-born male, delivered at term by cesarean section, with an uneventful perinatal period. There was no history of consanguinity between the parents. He had been exclusively breastfed since birth until 5 months of age, and there was no history of prolonged physiological jaundice, abdominal pain, vomiting, pancreatitis, failure to thrive, or hospitalizations. Family history was negative for premature cardiovascular disease, pancreatitis, or disorders of lipid metabolism.

Auxologic evaluation at 5 months of age revealed a length of 65 cm (25.6 inches; +0.34 SDS), weight of 6 kg (13.2 pounds; −1.15 SDS), body mass index (BMI) of 16.57 kg/m^2^ (−0.18 SDS), and head circumference of 42 cm (16.5 inches; +0.4 SDS). General physical examination was unremarkable. There were no eruptive xanthomas or dysmorphic features. There were no lipodystrophic features in the infant. The abdomen was soft and nontender, with no organomegaly. Systemic examination was unremarkable. Genital examination revealed Tanner stage I, with both testes in the scrotum. The infant was hemodynamically stable, with a blood pressure of 90/50 mm Hg, heart rate of 106 beats/min, respiratory rate of 20 breaths/min and was afebrile. Fundoscopic examination revealed bilateral lipemia retinalis (Fig. 2).

**Figure 2:**
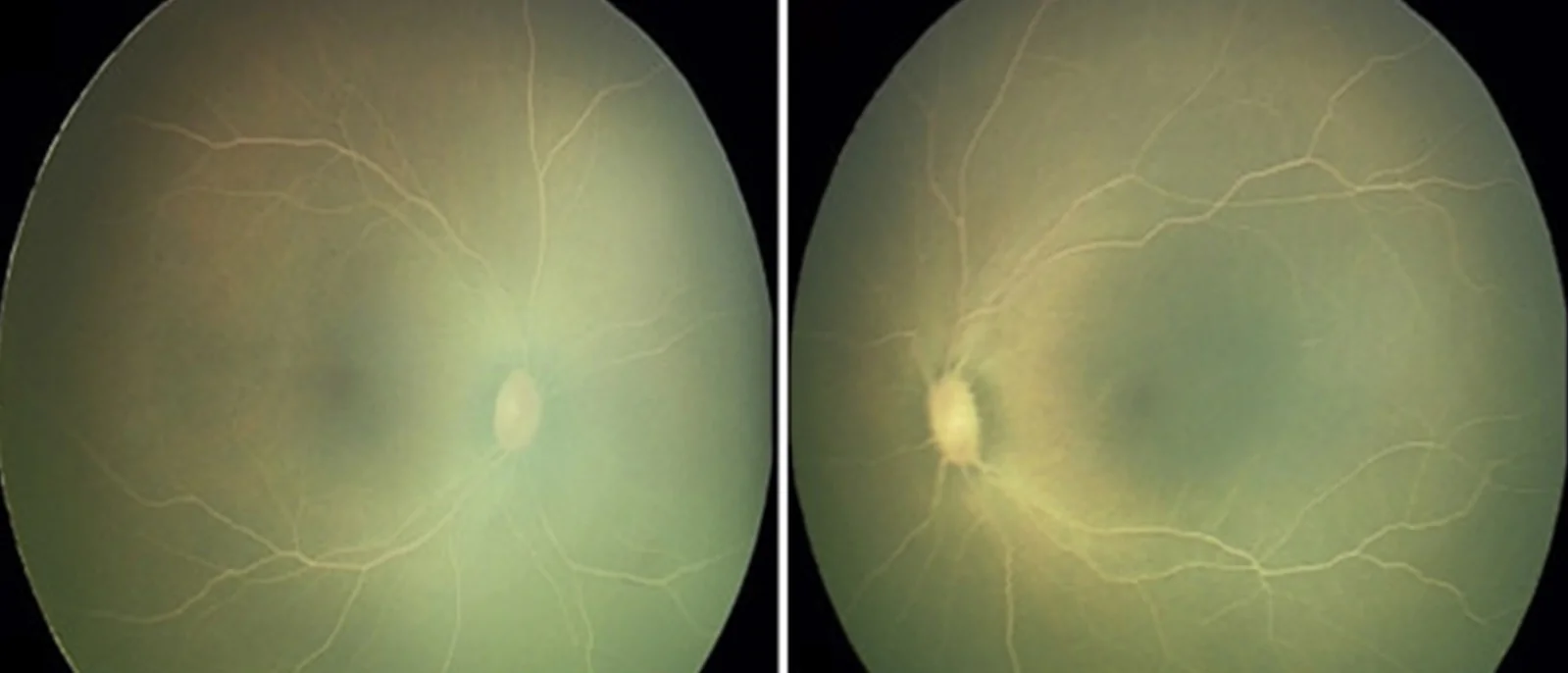
Bilateral lipemia retinalis. Color fundus photographs of both eyes showing diffuse creamy-white retinal discoloration, consistent with lipemia retinalis secondary to severe hypertriglyceridemia.

## Diagnostic assessment

Biochemical evaluation revealed serum TG levels of 8014 mg/dL (207.2 mmol/L) (reference range, <75 mg/dL [SI: <0.85 mmol/L]), which remained elevated on repeat testing. Serum total cholesterol, high-density lipoprotein cholesterol and low-density lipoprotein (LDL) cholesterol were 640 mg/dL (16.6 mmol/L) (reference range, <170 mg/dL [SI: <4.4 mmol/L]), 11 mg/dL (0.28 mmol/L) (reference range, >45 mg/dL [SI: >1.2 mmol/L]) and 10 mg/dL (0.26 mmol/L) (reference range, <110 mg/dL [SI: <2.8 mmol/L]), respectively. Lipids were measured using an automated biochemical analyzer (Siemens AUTOPAK 300). Serum LDL cholesterol was measured directly by using homogenous enzymatic method. Fasting plasma glucose was 78 mg/dL (4.3 mmol/L) (reference range, 70-99 mg/dL [SI: 3.9-5.5 mmol/L]) and 2-hour postprandial plasma glucose was 100 mg/dL (5.6 mmol/L) (reference range, 70-140 mg/dL [SI: 3.9-7.8 mmol/L]). Free thyroxine (fT4), free triiodothyronine (fT3), and thyroid-stimulating hormone were 1.36 ng/dL (17.5 pmol/L) (reference range, 0.8-1.8 ng/dL [SI: 10-23 pmol/L]), 2.98 pg/mL (4.6 pmol/L) (reference range, 2.3-4.2 pg/mL [SI: 3.5-6.5 pmol/L]) and 1.04 mIU/L (reference range, 0.4-4.0 mIU/L), respectively. Serum amylase and lipase were 23 IU/L (reference range, 30-110 IU/L) and 30 IU/L (reference range, 0-160 IU/L), respectively. Liver and renal function tests were within reference ranges. Ultrasonography of the abdomen and pelvis was normal with no evidence of pancreatitis. Estimation of lipid profiles of both parents were normal. Given the severity of hypertriglyceridemia and early age of onset, genetic testing was pursued.

Blood was collected in an ethylenediaminetetraacetic acid vial, and DNA was extracted for targeted gene capture using a custom kit. Sequences were aligned to the human reference genome (GRCh38) using the Burrows–Wheeler Aligner (Sentieon, PMID: 20080505). Variants were identified with Sentieon haplotype caller, and copy number variants were detected using ExomeDepth (PMID: 22942019) method. This identified a homozygous nonsense variant (p.Arg233Ter) in Exon 5 of the *LMF1* gene (chr16:g.893039G>A). This variant (p.Arg233Ter) results in a stop codon and premature truncation of the LMF1 protein at codon 233. This variant has been classified as pathogenic for combined lipase deficiency with hyperchylomicronemia in the ClinVar database.

## Treatment

At presentation, the infant was receiving ∼600 mL of breast milk daily, contributing nearly 23 g of fat per day (∼390 kcal/day, 50% of total caloric intake contributed by fat) [16-18], significantly exceeding the recommended fat intake for FCS [6]. Breastfeeding was discontinued, and the infant was transitioned to skimmed milk powder feeds containing medium-chain TG (MCT) oil. The infant's caloric requirement as per age was 600 kcal (100 kcal/kg/day) [19]. The skimmed milk powder (Simyl MCT) provides 482 kcal, 12 g of milk fat, and 7.4 g of MCT per 100 g. The patient was started on 85 g of Simyl MCT, divided into 5 feeds, providing ∼410 kcal, containing ∼140 kcal from fat. Due to the unavailability of completely fat-free infant formula in our setting, the rest of the caloric requirement was supplemented with sucrose (10 g per feed), providing an additional 190 kcal.

Each feed was diluted with 180 mL of water as advised by the manufacturer. Fat contributed ∼20% of the total caloric intake from these formula feeds. This feeding regimen was continued for 1 month, after which serum TG levels were re-estimated and had decreased from 8014 mg/dL (90.5 mmol/L) to 5040 mg/dL (56.9 mmol/L).

## Outcome and follow-up

At 6 months of age and a weight of 7 kg, weaning was started with rice- and vegetable-based diet, supplemented with MCT oil (Simyl MCT oil). Each milliliter of MCT oil provides 7.8 kcal (as per the manufacturer's label). This diet consisted of 120 mL of a traditional Indian preparation made from rice, lentils, and vegetables (khichdi) and 2 mL of MCT oil given 4 times daily (total: 480 kcal, 62.4 kcal from fat) [19]. The rest of the caloric requirement was met with one serving of mashed seasonal fruit supplemented with 1 mL of MCT oil given twice daily (total: 220 kcal). This diet provided ∼700 kcal, which was appropriate for his age and weight, and restricted fat intake to 11% of total daily calories. It was continued for 4 months, and by 10 months of age, fasting serum TG levels had decreased to 307 mg/dL (3.5 mmol/L). Repeat serum samples no longer demonstrated a lipemic layer (Fig. 1C). Growth and developmental milestones were appropriate for age, and there were no episodes of pancreatitis or abdominal pain during follow-up.

At the last follow-up, at 1 year and 6 months of age, he weighed 12 kg (+1.26 SDS), having gained 5 kg over 1 year. Other anthropometric parameters were appropriate for age. His serum TG level was 225 mg/dL (2.54 mmol/L), with no clinical signs of pancreatitis.

## Discussion

FCS is often diagnosed in adolescence or young adults, when they present with recurrent pancreatitis or eruptive xanthomas. Early diagnosis can lead to timely initiation of dietary and pharmacological therapy. FCS needs to be distinguished from multifactorial chylomicronemia syndrome, which is a polygenic disorder associated with obesity, type 2 diabetes mellitus, alcohol intake, hypothyroidism, or exposure to certain drugs [5]. FCS should also be distinguished from lipodystrophies and other monogenic disorders presenting with severe hypertriglyceridemia.

The management of FCS in infancy is challenging, and there are no approved pharmacological therapies for the same [6]. Dietary modification reducing the long-chain TG content of feeds, along with supplementation with oils containing MCT, is required for control of hypertriglyceridemia [6]. Treatment of FCS is mainly focused on reducing fat intake to <10% to 15% of total calories [6]. MCT oil contains MCT, which are directly absorbed into the portal vein and do not require LPL for metabolism [20].

LPL is ﬁrst synthesized in the rough endoplasmic reticulum of adipose tissue, heart, and skeletal muscles as an inactive monomer [21]. LMF1 is essential for folding, dimerization, secretion, and activation of LPL, as well as other related lipases, namely hepatic lipase and endothelial lipase [21]. A speciﬁc chaperone called Sel1L stabilizes the LPL-LMF1 complex [21]. LPL is transported and anchored to the luminal surface of endothelial cells by GPIHBP1 [20]. LPL-mediated lipolysis of TG is stimulated by APOC2 and APOA5 and inhibited by APOCI and APOC3 [21]. Homozygous or compound heterozygous loss-of-function variants of *LPL*, *APOC2*, *APOA5*, *GPIHBP1*, and *LMF1* can lead to FCS [5, 6].

Variants in the *LMF1* gene causing FCS are rare [5, 7-15]. Reported cases typically presented in early adulthood (23-65 years) and were characterized by recurrent episodes of pancreatitis, as shown in Table 1. In contrast, the present case is unique because FCS due to a homozygous nonsense variant (p.Arg233Ter) in *LMF1* was incidentally diagnosed during infancy. A similar variant was reported by Tanaka et al in a patient evaluated at 35 years of age [8]. To the best of our knowledge, this is the first reported case of FCS due to a homozygous *LMF1* variant presenting in infancy.

**Table 1:** Previously reported cases of FCS with biallelic variants in the *LMF1* gene

Age	Sex	*LMF1* variant	Clinical features	Serum TG levels	Reference range for serum TG	Authors
46 years	Male	Homozygous in-frame duplication in *LMF1* (c.914_928dup;p.Ser309_Phe310dup)	Eight episodes of pancreatitis since the age of 28 years with peak serum TG levels of 8900 mg/dL (100.5 mmol/L).	8900 mg/dL (100.5 mmol/L)	<150 mg/dL (1.7 mmol/L)	Vallejo et al (2026) [5]
23 years	Male	Homozygous in-frame duplication in *LMF1* (c.914_928dup; p.Ser309_Phe310dup)	Three episodes of pancreatitis, with peak serum TG levels of 7000 mg/dL (79.0 mmol/L).	7000 mg/dL (79.0 mmol/L)	<150 mg/dL (1.7 mmol/L)	Vallejo et al (2026) [5]
26 years	Female	Homozygous in-frame duplication in *LMF1* (c.914_928dup; p.Ser309_Phe310dup)	Four episodes of pancreatitis since the age of 23 years with serum TG levels of 1233 mg/dL (13.9 mmol/L).	1233 mg/dL (13.9 mmol/L)	<150 mg/dL (1.7 mmol/L)	Vallejo et al. (2026) [5]
35 years	Males	Compound heterozygous variant (c.504G>A/p.W168X and c.1247G>A/p.R416Q) in exons 3 and 9 of *LMF1*	Several episodes of acute pancreatitis since the age of 25 years with highest serum TG levels of 2993 mg/dL (33.8 mmol/L).	2993 mg/dL (33.8 mmol/L)	<150 mg/dL (1.7 mmol/L)	Cao et al (2024) [7]
35 years	Female	Homozygous nonsense variant (p.Arg233Ter) in Exon 5 of *LMF1* gene	HTG was diagnosed at 8 years of age, managed with dietary therapy, with exacerbation at 32 weeks of pregnancy, with serum TG levels of 4429 mg/dL (>50 mmol/L).	4429 mg/dL (>50 mmol/L)	<150 mg/dL (1.7 mmol/L)	Tanaka et al (2023) [8]
30 years	Female	Homozygous variant: c.897G>A (p.Gln299=), near splice site between exon 6 and intron 6	HTG since the age of 10 years with highest serum TG of 1125 mg/dL (12.7 mmol/L) and repeated episodes of pancreatitis.	1125 mg/dL (12.7 mmol/L)	<150 mg/dL (1.7 mmol/L)	Garay-García et al (2022) [9]
23 years	Female	Homozygous variant: c.897G>A (p.Gln299=), near splice site between exon 6 and intron 6.	HTG since the age of 18 years with highest serum TG of 1200 mg/dL (13.6 mmol/L) and repeated episodes of abdominal pain.	1200 mg/dL (13.6 mmol/L)	<150 mg/dL (1.7 mmol/L)	Garay-García et al (2022) [9]
46 years	Male	Homozygous missense variant in *LMF1* gene: c.233T>C (p.Leu78Pro), chr16:954 627 A>G	HTG since childhood with serum TG of 5293 mg/dL (59.7 mmol/L) and history of pancreatitis.	5293 mg/dL (59.7 mmol/L)	<150 mg/dL (1.7 mmol/L)	Garay-García et al (2022) [9]
65 years	Male	Homozygous loss-of-function variant in *LMF1* gene: p.Gly172Arg *LMF1*	HTG diagnosed at 30 years with highest serum TG of 4429 mg/dL (50 mmol/L) with one episode of pancreatitis at 35 years.	4429 mg/dL (50mmol/L)	<150 mg/dL (1.7 mmol/L)	Serveaux et al (2018) [10]
51 years	Male	Compound heterozygous variants in *LMF1* gene (c.257C>T/p.P86L and c.1184C>T/p.T395I)	HTG with serum TG levels of 3427 mg/dL (38.7 mmol/L) and history of one episode of pancreatitis.	3427 mg/dL (38.7 mmol/L)	<150 mg/dL (1.7 mmol/L)	Liu et al (2019) [11]
44 years	Male	Compound heterozygous variants in *LMF1* gene: c.157delC (p.Arg53Glyfs*5) and c.410C>T (p.Ser137Leu).	Serum TG 2000 mg/dL (22.6 mmol/L) at 36 years of age, pancreatitis at 44 years of age.	2000 mg/dL (22.6 mmol/L)	<150 mg/dL (1.7 mmol/L)	Péterfy et al (2018) [12]
54 years	Male	Homozygous nonsense variant in *LMF1* gene (c.1380C>G, p.Y460X).	Serum TG levels of 2500 mg/dL (28.2 mmol/L)	2500 mg/dL (28.2 mmol/L)	<150 mg/dL (1.7 mmol/L)	Cefalu et al (2017) [13]
42 years	Male	Homozygous G>A substitution in exon 9 of *LMF1* gene	Two episodes of pancreatitis since the age of 32 years with serum TG levels of 2400 mg/dL (27.1 mmol/L).	2400 mg/dL (27.1 mmol/L)	<150 mg/dL (1.7 mmol/L)	Cefalu et al (2009) [14]
48 years	Female	Homozygous C>G nonsense variant (nucleotide 1319 in NM_022773) in exon 9 of *LMF1* gene	Several episodes of pancreatitis with serum TG levels of 2500-3000 mg/dL (28.2-33.9 mmol/L).	2500-3000 mg/dL (28.2-33.9 mmol/L)	<150 mg/dL (1.7 mmol/L)	Cao et al (2024) [7] and Péterfy et al (2007) [15]
5 months	Male	Homozygous nonsense variant (p.Arg233Ter) in Exon 5 of *LMF1* gene	Incidentally detected serum TG levels of 8014 mg/dL (207.2 mmol/L), during evaluation for lipemic serum.	8014 mg/dL (207.2 mmol/L)	<100 mg/dL (1.13 mmol/L)	

Abbreviations: *LMF1*, lipase maturation factor 1; TG, triglyceride; HTG, hypertriglyceridemia.

## Learning points

- FCS due to a *LMF1* variant is an exceptionally rare cause of severe hypertriglyceridemia presenting in infancy.
- Incidental observation of lipemic serum may be the earliest diagnostic clue, preceding overt clinical manifestations, and should prompt immediate assessment of serum TG levels in infants.
- Early recognition, a high index of suspicion, and timely genetic confirmation facilitate initiation of targeted dietary therapy.
- Strict fat restriction with MCT supplementation can result in marked biochemical improvement and prevention of pancreatitis.

## Contributors

All authors made individual contributions to authorship. P.R.K., B.D., D.K.D., D.P., P.K.S., and A.B. were involved in the diagnosis, management, and manuscript submission. All authors reviewed and approved the final draft.

## Data Availability

Some or all datasets generated during and/or analyzed during the current study are not publicly available but are available from the corresponding author on reasonable request.
